# Empirical Support for DSM-IV Schizoaffective Disorder: Clinical and Cognitive Validators from a Large Patient Sample

**DOI:** 10.1371/journal.pone.0063734

**Published:** 2013-05-30

**Authors:** Pamela DeRosse, Katherine E. Burdick, Todd Lencz, Samuel G. Siris, Anil K. Malhotra

**Affiliations:** 1 Center for Translational Psychiatry, The Feinstein Institute for Medical Research, Manhasset, New York, United States of America; 2 Division of Psychiatry Research, The Zucker Hillside Hospital, Division of the North Shore–Long Island Jewish Health System, Glen Oaks, New York, United States of America; 3 Departments of Psychiatry and Neuroscience at Mount Sinai School of Medicine, New York, New York, United States of America; 4 Hofstra North Shore LIJ School of Medicine, Hempstead, New York, United States of America; Chiba University Center for Forensic Mental Health, Japan

## Abstract

**Objective:**

The diagnosis of schizoaffective disorder has long maintained an uncertain status in psychiatric nosology. Studies comparing clinical and biological features of patients with schizoaffective disorder to patients with related disorders [e.g., schizophrenia and bipolar disorder] can provide an evidence base for judging the validity of the diagnostic category. However, because most prior studies of schizoaffective disorder have only evaluated differences between groups at a static timepoint, it is unclear how these disorders may be related when the entire illness course is taken into consideration.

**Methods:**

We ascertained a large cohort [N = 993] of psychiatric patients with a range of psychotic diagnoses including schizophrenia with no history of major affective episodes [SZ**−**; N = 371], schizophrenia with a superimposed mood syndrome [SZ+; N = 224], schizoaffective disorder [SAD; N = 129] and bipolar I disorder with psychotic features [BPD+; N = 269]. Using cross-sectional data we designed key clinical and neurocognitive dependent measures that allowed us to test longitudinal hypotheses about the differences between these diagnostic entities.

**Results:**

Large differences between diagnostic groups on several demographic and clinical variables were observed. Most notably, groups differed on a putative measure of cognitive decline. Specifically, the SAD group demonstrated significantly greater post-onset cognitive decline compared to the BP+ group, with the SZ− and SZ+ group both exhibiting levels of decline intermediate to BPD+ and SAD.

**Conclusions:**

These results suggest that schizoaffective disorder may possess distinct features. Contrary to earlier formulations, schizoaffective disorder may be a more severe form of illness.

## Introduction

Since its initial description by Kasanin [1] the diagnosis of schizoaffective disorder [SAD] has been at the center of controversy regarding the relation between psychotic and affective disorders. Originally described as a disorder in which patients presented with concomitant and equally severe affective and psychotic symptoms, SAD was conceptualized as a “good outcome” sub-type of schizophrenia (SZ) [2], [3]. It was not until the publication of the 3^rd^ edition of the Diagnostic and Statistical Manual (DSM-III) of the American Psychiatric Association that SAD became a separate and more refined diagnostic entity and strict operational criteria for the diagnosis of SAD were not described until the publication of the DSM-IIIR [4]. Unfortunately, these diagnostic criteria are not without ambiguities.

For example, according to current diagnostic criteria (DSM-IV-TR) [5], a patient with symptoms meeting the diagnostic criteria for both SZ and a manic episode may receive a diagnosis of either SZ with bipolar disorder NOS (BPD NOS), SAD or bipolar disorder (BPD) with psychotic features. The critical distinction in the differential diagnosis is the relative time course of psychotic and affective symptoms: if psychotic symptoms are not present for at least 2 weeks in the absence of manic symptoms, a diagnosis of BPD with psychotic features is warranted; if psychotic symptoms are present for at least 2 weeks in the absence of manic symptoms AND manic symptoms are “relatively brief” in comparison to the total duration of illness then a diagnosis of SZ with BPD NOS is warranted. If none of these criteria are met, a diagnosis of SAD is then appropriate. For this reason, SAD has often been referred to as a “diagnosis by exclusion” [6].

Differential diagnosis based on the temporal relationship between psychotic and affective symptoms is often problematic at the clinical level. Because the relative proportion of affective and psychotic symptoms may change over the course of illness, a diagnosis of SAD may be warranted at a cross-sectional level during an acute episode whereas a diagnosis of SZ with BPD NOS may be warranted at the longitudinal level. Consistent with this hypothesis, Schwartz et al [7] found that the concordance of diagnoses at baseline vs. 24-month follow up was relatively high for both SZ and affective disorders (91.72% and 82.98%, respectively) but low for SAD (36.36%) in a study of 548 patients presenting with their first episode of psychosis; of those diagnosed with SAD at baseline, 42.24% switched to SZ and 21.21% switched to affective disorders at the 24-month follow up.

In addition to differences in illness course, clinical and neurocognitive variables may also differentiate SAD from SZ and psychotic BPD. Studies seeking to elucidate differences among these groups, however, have reported mixed results. For example, while some studies have reported differences in levels of negative [8], [9], disorganized [9] and positive symptoms [10] among SZ, SAD and BPD groups, others have reported no differences in negative [11], [10], disorganized [11] and positive symptoms [8], [9]. Assessment of differences in neurocognitive variables have also produced mixed findings (see [12] for a comprehensive review). Some studies suggest that the cognitive impairment in SAD is similar to SZ and worse than BPD while others have suggest that the cognitive impairment in SAD is similar to BPD and better than SZ. Yet others have suggested that the cognitive impairment in SAD is intermediate to those of SZ and BPD. Such conclusions, however, have been drawn primarily from cross-sectional studies that have focused on differences between the diagnostic groups at a static time point. Although prospective, longitudinal studies of first-episode psychosis have repeatedly found that cognitive decline during the early stages of illness predict conversion to SZ but not to BPD [13], [14], [15], [16], little effort has been directed at elucidating the cognitive trajectory (i.e. the change from premorbid to current cognitive function) of SAD.

Because SAD is a diagnosis that is dependent on the course of the illness, the utilization of dependent measures that cover the entire illness course may substantially enhance our ability to detect clinical features that differentiate SAD from SZ and psychotic BPD [17]. Thus, the present study evaluated differences in clinical features, including affective and psychotic symptoms, manifested over the entire course of illness in diagnostic groups including SZ without a concomitant mood syndrome, SZ with a concomitant mood syndrome, SAD and psychotic bipolar disorder. Moreover, because cognitive trajectory during the course of illness appears to reliably differentiate between SZ and affective disorders [18], we also sought to explore differences in cognitive trajectories that might exist among these diagnostic groups.

## Methods

### Sample

The sample included 993 subjects recruited from the inpatient and outpatient clinical services of the Zucker Hillside Hospital, a division of the North Shore–Long Island Jewish Health System, where patients are screened for potential recruitment into research studies by the Clinical Assessment and Training (CAT) unit of the National Institutes of Health–funded Hillside Hospital Intervention Research Center. The CAT unit monitors the inpatient and outpatient hospital census daily and conducts preliminary screening and recruitment functions. Inclusion criteria for this study included a clinical diagnosis of a psychotic disorder, no active substance abuse, fluency in the English language, and the ability to provide informed consent. All subjects provided written informed consent to a protocol approved by the Institutional Review Board of the North Shore-Long Island Jewish Health System. The total sample was divided based on DSM-IV diagnoses and included 1] schizophrenia with no history of major affective episodes (SZ−; N = 371), 2) schizophrenia with a superimposed mood syndrome (depressive disorder NOS or bipolar disorder NOS) (SZ+; N = 224), 3) schizoaffective disorder (SAD; N = 129) and 4) bipolar I disorder with psychotic features (BPD+; N = 269).

### Clinical Assessment

Each subject was assessed with the Structured Clinical Interview for the DSM-IV (SCID), which was administered by trained and reliable raters. Information obtained from the SCID was supplemented by a comprehensive review of all available medical records and when possible, interviews with family informants. Information derived from the interview along with information from the medical record and family informants, were utilized to rate the SCID. The SCID data were then compiled into a narrative clinical case summary describing the patient's entire illness course. Diagnoses were then determined by a consensus among a minimum of three expert diagnosticians from the ZHH faculty. We have also operationalized DSM–IV Criterion C for schizoaffective disorder, which differentiates it from schizophrenia. Criterion C requires that mood symptoms meeting criteria for a mood episode ‘must be present for a substantial portion of the entire period of illness' for a diagnosis of schizoaffective disorder to be made. Our operationalized criterion defined ‘a substantial portion’ as greater than 30% of the total duration of illness.

Because symptom severity often varies during the course of illness, a lifetime symptom severity rating rather than a cross-sectional rating was used [17], [19]. Lifetime symptom ratings were derived from SCID data and included overall ratings on two categories of symptoms: 1) psychotic symptoms including negative symptoms, positive symptoms and disorganized symptoms and 2) affective symptoms including depressive and manic symptoms. Individual psychotic symptom ratings were obtained by summing the scores for each symptom within a domain such that the negative symptom rating included ratings on avolition, alogia and affective flattening [20], the positive symptom rating included ratings on delusions (referential, paranoid, grandiose, somatic, control, thought broadcasting, bizarre, and other delusions) and hallucinations (auditory, visual, tactile and other hallucinations) [21] and the disorganization symptom rating included ratings on disorganized speech and disorganized behavior [22]. The total psychotic symptom rating was produced by summing all of these categories. Individual affective symptom ratings were also obtained by summing the scores for each symptom within a domain such that manic symptoms rating included ratings on elevated, euphoric or irritable mood, inflated self-esteem, decreased need for sleep, pressured speech or talkativeness, flight of ideas or racing thoughts, distractibility, increase in activity and impulsivity [23] and the depressive symptom rating included ratings on depressed mood, anhedonia, weight or appetite disturbance, sleep disturbance, psychomotor retardation or agitation, energy disturbance, worthlessness or inappropriate guilt, diminished concentration or difficulty making decisions and suicidal thoughts or behavior. The total affective symptom rating was produced by summing the overall manic and depressive scores.

Ratings on each of the items were recorded based on the subject's report during the interview as well as the medical record and other available sources and are rated on a continuous scale where 1 = absent, 2 = subthreshold and 3 = present. All symptoms were rated based on a lifetime history. Because affective symptoms may be rated for both present and past mood episodes, the affective symptoms used to create the composite score were computed using whichever section (present or past) yielded the higher score.

### Neurocognitive Measures

Participants were administered a battery of standardized cognitive measures comprised of the Wechsler Adult Intelligence Test-Revised (WAIS-R)-Digit Span; California Verbal Learning Test (CVLT)-Abridged; Controlled Oral Word Association Test (COWAT), fluency (animal naming), and Trail Making Tests A & B. Because our interest was primarily in lifetime illness course, simple comparisons among groups on these neurocognitive measures would not be informative. Thus, we sought to assess the change in neurocognitive function over time across diagnostic groups. Because longitudinal data were not available we used an alternative approach to generate indices of cognitive trajectory over the course of illness.

Following common practice in the psychiatric literature [24], we estimated premorbid IQ using the Wide Range Achievement Test-Third Edition-Reading Subtest (WRAT-3). WRAT-3 is a test that assesses single word reading skill which, like command of general knowledge and vocabulary, is particularly resistant to the effects of deterioration associated with brain disease and is considered a reliable estimate of pre-morbid IQ in patient populations [25].

As a proxy for current IQ we calculated “general cognitive ability”, or (*g*), as the first component of an unrotated principal components analysis utilizing all of the cognitive tests administered, except the WRAT-3. A detailed description of the PCA methods used to derive *g* is provided in Burdick et al. [26]. Briefly, all cognitive variable data were transformed to standardized z-scores and missing values were replaced by the mean of the group. No case with more than two missing values was retained in the sample and a formal missing value analysis was conducted to rule out any consistent patterns in missing values. A single factor model was produced (extracted variables with eigenvalues of >1.0 using the regression method). This first unrotated factor explained 51.7% of the variance and represented a general cognitive ability factor. Each of the individual measures loaded onto the first factor with covariance of >0.64.

### Statistical Analyses

#### Demographic variables

Comparison of demographic variables including sex, race and family history of psychotic illness were carried out using chi square analyses. Additional analyses of demographic variables including age, age at onset of psychiatric illness, duration of psychiatric illness and global assessment of functioning score were carried out using a series of one-way ANCOVAs that covaried for any differences observed in sex, race or family history of psychotic illness.

#### Symptom dimensions

To evaluate the differences between diagnostic groups on psychotic and affective symptom clusters, ANCOVA's were carried out to compare diagnostic groups on lifetime severity of psychotic and affective symptoms. In these analyses, all of the demographic variables that were shown to differ between groups were entered into the model as covariates. Significant results were followed up with post-hoc analyses to determine the specific differences among groups.

#### Neurocognitive measures

Consistent with methodology used in Kremen et al. [25] we measured deviations from premorbid IQ by using a regression approach. This method is preferable to using raw score differences because the premorbid-current discrepancy may not be equivalent at all IQ levels. This was done by regressing *g* on WRAT-3 in a healthy control sample matched on sex, age and race (n = 145) and using the resulting regression equation to generate standardized scores, representing estimated premorbid IQ, in each of the diagnostic groups. Thus, the putative measure of cognitive trajectory described is actually the discrepancy between current IQ, as measured by *g*, and estimated premorbid IQ based on a predicted score [14].

To evaluate differences in the cognitive trajectories of the diagnostic groups we carried out a repeated-measure ANOVA where the within-subjects factor represented our putative cognitive trajectory [estimated premorbid IQ derived from WRAT regression vs. current IQ estimated from g] and diagnostic group represented the between-subjects factor. However, because cognitive trajectories are likely to vary as a function of demographic characteristics and level of clinical symptomatology, this analysis was followed-up with a repeated-measure ANCOVA that included these variables as covariates.

## Results

### Demographic variables

Comparison of demographic variables amongst the groups revealed a significant difference between groups on sex distribution with fewer females in the both the SZ− and SZ+ groups relative to the SAD group and BPD+ groups. Differences in racial composition were also observed between groups with more non-whites in the SZ− group relative to both the SAD and BPD+ groups and more non-whites in the SZ+ group relative to the BPD+ group. Moreover, diagnostic groups also differed in the family history of psychotic illness with the BPD+ group being more likely to have a family history of psychotic illness than the SZ− group. A series of ANCOVA's, which covaried for all three of these factors, were carried out to assess differences in diagnostic group on age, age at onset of psychiatric illness, duration of psychiatric illness and GAF score. These analyses revealed significant differences between groups on age, with post-hoc analyses indicating that the SZ+ group, on average, was older than the both the SZ− and BPD+ groups. Significant differences between groups was also noted for illness duration with post-hoc tests indicating that the SZ+ group had a longer illness duration than the BPD+ group. Finally, differences between groups were also observed on GAF score with the BPD+ group having higher functioning than any of the other 3 groups and the SZ+ group higher than the SZ− group. No differences were observed between groups on age at onset of psychiatric illness. All of these results are shown in Table 1.

**Table 1:** Demographic characteristics of study sample: schizophrenia without mood syndrome [SZ−]; schizophrenia with superimposed mood syndrome [SZ+]; schizoaffective disorder [SAD]; bipolar disorder with psychotic features [BPD+].

	SZ− [N = 371]	SZ+ [N = 224]	SAD [N = 129]	BPD+ [N = 269]	Statistic	p value	Pairwise Comparisons
**Mean Age [SEM]**	37.09 [11.10]	39.18 [10.89]	37.93 [11.68]	36.69 [12.22]	F = 4.22	0.006	A, E
**Mean Age at Onset [SD]**	21.95 [5.87]	21.07 [6.37]	22.41 [7.11]	22.29 [8.12]	F = 2.31	NS	N/A
**Mean Illness Duration [SD]**	15.29 [10.80]	17.95 [10.84]	15.57 [10.75]	14.30 [11.06]	F = 5.86	0.001	E
**Mean GAF Score [SD]**	35.91 [14.15]	39.98 [14.10]	39.43 [14.07]	47.31 [16.44]	F = 10.89	<0.0001	A, C, E, F
**% Female**	26.15%	29.46%	55.81%	50.93%	X^2^ = 65.76	<0.0001	B, C, D, E
**% Family History**	18.70%	26.63%	24.27%	30.71%	X^2^ = 8.19	0.042	C
**Race [% White]**	40.43%	46.87%	56.59%	64.68%	X^2^ = 39.79	<0.0001	B, C, E

**Pairwise Comparisons:**

A  =  SZ− vs. SZ+.

B  =  SZ− vs. SAD.

C  =  SZ− vs. BPD+.

D  =  SZ+ vs. SAD.

E  =  SZ+ vs. BPD+.

F  =  SAD vs BPD+.

See text for additional details.

### Symptom dimensions

**Figure 1:**
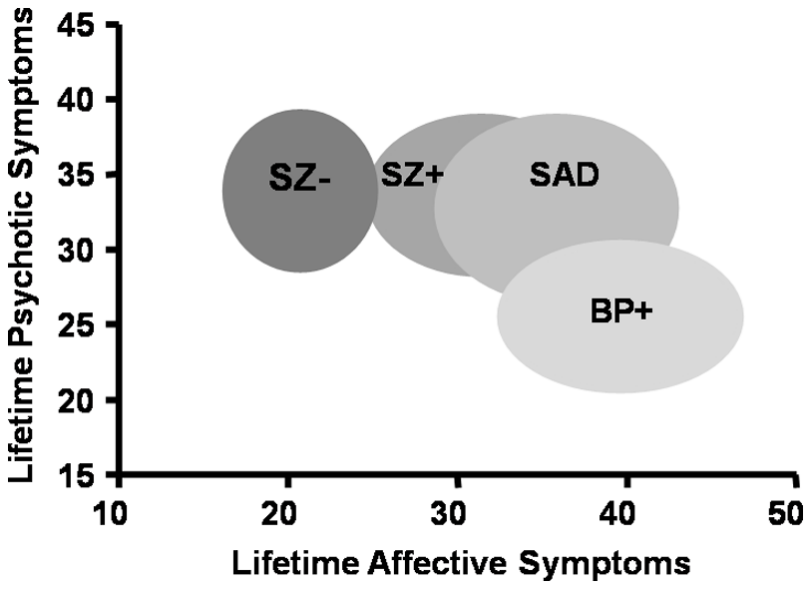
Dimensional representation of psychotic and affective symptoms across diagnostic groups. The boxes used to identify the groups encompass the mean of each group as well as their standard deviations on both dimensions: schizophrenia without mood syndrome [SZ−]; schizophrenia with superimposed mood syndrome [SZ+]; schizoaffective disorder [SAD]; bipolar disorder with psychotic features [BPD+].

To evaluate the differences between diagnostic groups on psychotic and affective symptom clusters, ANCOVA's were carried out using ratings of lifetime severity of psychotic and affective symptoms. In these analyses, sex, race, family history of psychotic illness, age, illness duration and GAF score were included as covariates. Diagnostic groups differed on both overall psychosis as well as overall affective symptoms. Post hoc analyses on lifetime severity of psychotic symptoms indicated that the BPD+ group had significantly less severe psychosis than any of the other 3 groups. No differences in psychotic symptoms were noted between the SZ+, SZ− and SAD groups. Post hoc analyses on lifetime severity of affective symptoms indicated that lifetime severity of affective symptoms were significantly different for all pairwise comparisons with a gradation in severity across diagnostic categories. Specifically, the SZ− group had the lowest overall rating, followed by the SZ+ group and then the SAD group and finally the BPD+ group. These data are illustrated in Figure 1.

Exploratory analyses were conducted to assess differences in diagnostic groups based on the specific symptom domains contained within the overall psychotic and affective symptom composite scores (e.g.: negative symptoms, manic symptoms, etc.). These analyses included the same covariates as the analyses conducted on the overall composite scores. The overall model was significant with significant differences being observed on all of the symptom domains. Post hoc analyses indicated that lifetime severity of mania was significantly different for all pairwise comparisons with a gradation in severity across diagnostic categories. Specifically, the SZ− group had the lowest overall rating, followed by the SZ+ group and then the SAD group and finally the BPD+ group. For lifetime severity of depressive symptoms, only the SZ− group differed from the other groups with the SZ+, SAD and BPD+ groups showing comparable levels of depressive symptomatology. For lifetime severity of disorganized, delusional and hallucination symptoms, only the BPD+ group differed from the others with significantly lower scores on these dimensions than either the SZ−, SZ+ or SAD groups. For the lifetime severity of negative symptoms, The BPD+ group had significantly lower ratings than any of the other groups and the SZ+ and SAD groups had lower ratings than the SZ− group. No difference was observed on negative symptoms for the SZ+ and SAD groups. All of these results are shown in Table 2.

**Table 2:** Mean symptom ratings and standard deviations for diagnostic groups: schizophrenia without mood syndrome [SZ−]; schizophrenia with superimposed mood syndrome [SZ+]; schizoaffective disorder [SAD]; bipolar disorder with psychotic features [BPD+].

	SZ− (N = 371)	SZ+ (N = 224)	SAD (N = 129)	BPD+ (N = 269)	Statistic	p value	Pairwise Comparisons
Hallucinations (SD)	6.79 (1.70)	7.03 (1.89)	6.89 (1.98)	5.57 (1.70)	*F = 17.29*	*<0.0001*	*C, E, F*
Auditory Hallucinations	2.71 (0.67)	2.68 (0.73)	2.55 (0.83)	1.70 (0.92)			
Visual Hallucinations	1.60 (0.85)	1.59 (0.85)	1.67 (0.88)	1.30 (0.66)			
Tactile Hallucinations	1.33 (0.71)	1.47 (0.79)	1.49 (0.81)	1.15 (0.48)			
Other Hallucinations	1.15 (.50)	1.26 (0.64)	1.29 (0.69)	1.14 (0.48)			

Delusions (SD)	16.00 (3.70)	16.65 (3.55)	15.99 (3.71)	12.84 (3.45)	*F = 16.72*	*<0.0001*	*C, E, F*
Referential Delusions	2.44 (0.80)	2.52 (0.76)	2.36 (0.86)	2.02 (0.92)			
Persecutory Delusions	2.61 (0.73)	2.58 (0.75)	2.49 (0.84)	1.78 (0.92)			
Grandiose Delusions	2.02 (0.96)	2.17 (0.95)	2.29 (0.93)	2.09 (0.96)			
Somatic Delusions	1.55 (0.86)	1.49 (0.83)	1.51 (0.86)	1.16 (0.47)			
Other Delusions	1.79 (0.94	1.78 (0.95)	1.98 (0.98)	1.66 (0.92)			
Control Delusions	1.86 (0.97)	1.91 (0.99)	1.67 (0.94)	1.25 (0.63			
Thought Broadcasting	1.67 (0.93	1.89 (0.98)	1.82 (0.98)	1.28 (0.65)			
Bizarre Delusions	2.33 (0.93)	2.25 (0.97)	2.20 (0.97)	1.33 (0.74)			

Negative Symptoms (SD)	5.73 (2.17)	4.83 (1.99)	4.48 (1.95)	3.45 (1.03)	*F = 24.45*	*<0.0001*	*A, B, C, D, E, F*
Avolition	2.08 (0.91)	1.73 (0.90)	1.50 (0.83)	1.07 (0.31)			
Alogia	1.85 (0.90)	1.59 (0.84)	1.40 (0.75)	1.05 (0.30)			
Affective Flattening	1.93 (0.84)	1.75 (0.85)	1.64 (0.83)	1.12 (0.38)			

Disorganized Symptoms (SD)	5.10 (1.85)	4.82 (1.82)	4.79 (1.82)	3.87 (1.38)	*F = 7.40*	*0.004*	*C, E, F*
Disorganized Behavior	1.91 (0.95)	1.81 (0.93)	1.70 (0.93)	1.29 (0.67)			
Disorganized Speech	1.91 (0.92)	1.81 (0.92)	1.88 (0.90)	1.33 (0.69)			

Depressive Symptoms (SD)	10.91 (3.69)	17.32 (5.91)	18.74 (5.76)	18.98 (5.84)	*F = 63.95*	*<0.0001*	*A, B, C*
Depressed Mood	1.23 (0.56)	1.55 (0.88)	1.85 (0.97)	1.57 (0.88)			
Anhedonia	1.46 (076)	2.33 (0.90)	2.46 (0.89)	2.52 (0.82)			
Weight/Appetite Change	1.14 (0.47)	1.88 (0.96)	1.92 (0.97)	2.19 (0.94)			
Sleep Disturbance	1.22 (0.58)	2.11 (0.98)	2.25 (0.95)	2.34 (0.92)			
Psychomotor Agitation/Retardation	1.15 (0.47)	1.81 (0.92)	1.98 (0.96)	1.94 (0.96)			
Energy Loss or Fatigue	1.22 (0.60)	2.02 (0.99)	2.20 (0.95)	2.34 (0.92)			
Feelings of Guilt or Worthlessness	1.17 (0.53)	1.86 (0.95	2.12 (0.96)	2.24 (0.92)			
Concentration/Thinking Difficulties	1.21 (0.59)	1.92 (0.97)	2.13 (0.95)	2.19)0.95)			
Suicidality	1.16 (0.52)	1.88 (0.98)	1.92 (0.98)	1.88 (0.98)			

Manic Symptoms (SD)	9.77 (2.11)	14.03 (5.23)	17.10 (5.22)	20.60 (3.05)	*F = 146.91*	*<0.0001*	*A, B, C, D, E, F*
Manic/Irritable Mood	1.34 (0.71)	2.16 (0.98)	2.52 (0.85)	3.00 (0)			
Grandiosity	1.14 (0.48)	1.79 (0.96)	2.12 (0.98)	2.61 (0.76)			
Decreased Sleep	1.08 (0.37)	1.67 (0.92)	2.05 (0.96)	2.63 (0.74)			
Pressured Speech	1.10 (0.39)	1.71 (0.94)	2.12 (0.98)	2.60 (0.75)			
Flight of Ideas	1.08 (0.39)	1.70 (0.93)	2.13 (0.99)	2.50 (0.83)			
Distractability	1.05 (0.29)	1.35 (0.73)	1.67 (0.93)	2.33 (0.91)			
Increase in Goal Behavior	1.12 (0.45)	1.78 (0.95)	2.19 (0.96)	2.70 (0.67)			
Impulsivity	1.05 (0.26)	1.55 (0.86)	2.01 (0.98)	2.39 (0.87)			

**Pairwise Comparisons.**

A  =  SZ− vs. SZ+.

B  =  SZ− vs. SAD.

C  =  SZ− vs. BPD+.

D  =  SZ+ vs. SAD.

E  =  SZ+ vs. BPD+.

F  =  SAD vs BP+.

See text for additional details.

### Neurocognitive measures

**Figure 2:**
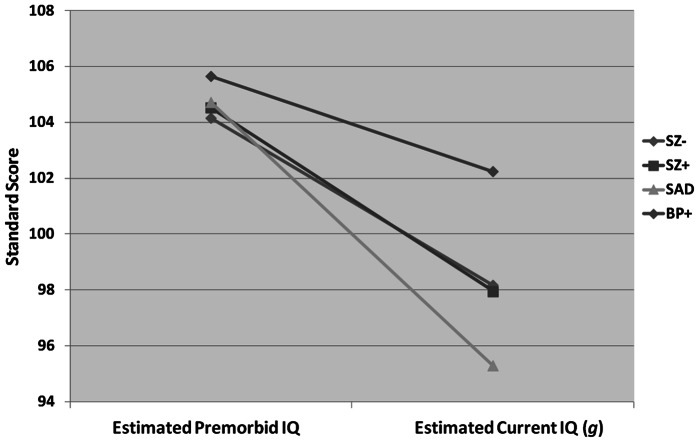
Cognitive decline by diagnostic group. Data are presented as residual scores as opposed to raw scores. Means have been adjusted for differences in demographic factors as well as lifetime symptom severity between groups: schizophrenia without mood syndrome [SZ−]; schizophrenia with superimposed mood syndrome [SZ+]; schizoaffective disorder [SAD]; bipolar disorder with psychotic features [BPD+].

To evaluate differences in cognitive trajectories between diagnostic groups we carried out a repeated-measures ANOVA where cognitive decline, measured by the discrepancy between our estimate of premorbid IQ and our proxy of current IQ (*g*), represented the within-subjects factor and diagnostic group the between-subjects factor. This analysis yielded a significant cognitive decline x diagnostic group interaction (F = 7.33; df = 3,629; p<0.0001); a significant main effect of diagnostic group (F = 12.54; df = 3, 629; p<0.0001) was also observed but was better accounted for by the interaction effect (see Figure 2). Because cognitive decline is likely to vary as a function of both demographic characteristics and level of clinical symptomatology, this analysis was followed-up with a repeated-measures ANCOVA that included sex, race, age, illness duration, GAF score as well as the scores on all symptom domains (delusions & hallucinations, negative, disorganized, manic and depressive symptoms) as covariates. This analysis yielded similar results indicating a significant cognitive decline x diagnostic group interaction (F = 3.07; df = 3,438; p = 0.03) as well as a significant main effect of group (F = 2.61; df = 3,438; p = 0.05). Significant covariates in this model included age (p = 0.04), race (p<0.0001) and depressive symptoms (p = 0.007). Post-hoc tests, corrected for multiple testing using a Bonferroni correction, indicated that the SAD group significantly differed from the BPD+ group on the putative measure of cognitive decline (p = 0.03). No other differences between diagnostic groups were observed. These data are illustrated in Figure 2.

## Discussion

The results of the present analyses suggest that there are several clinical characteristics of SAD that are more consistent with the SZ diagnostic groups and several others that are more consistent with the BPD+ group. Consistent with previous literature [27], the SAD group was similar to the BPD+ group [and different from both the SZ− and SZ+ groups] on several demographic characteristics including race and sex composition. In regards to lifetime severity of psychotic symptoms, the SAD group did not differ significantly from the SZ− and SZ+ group indicating comparable levels of psychosis across these diagnostic groups. Upon further analysis, however, the observed similarities in psychotic symptoms across the SZ−, SZ+ and SAD groups was limited to the positive dimensions of psychosis (hallucinations and delusions). In terms of the negative symptoms of psychosis (avolition, alogia and flat affect), all of the groups were significantly different from one another with the SZ− group showing the highest level of negative symptoms followed by the SZ+ group, the SAD group and the BPD+ group.

Comparison of groups on lifetime severity of affective symptoms also indicated significant differences between all of the groups with the BPD+ group showing the highest level of affective symptoms followed by the SAD group, the SZ+ group and SZ− group. Upon further analysis, however, the observed differences in affective symptoms were primarily limited to the lifetime severity of manic symptoms. On measures of lifetime severity of manic symptoms, all of the groups were significantly different from one another with the BPD+ group having the highest rating, followed by the SAD group, the SZ+ group and the SZ− group. On measures of lifetime severity of depressive symptoms, however, only the SZ− group differed from the other diagnostic groups with the SZ+, SAD and BPD+ groups showing comparable levels of depressive symptomatology over the illness course.

At the clinical level it is often difficult to differentiate between negative symptoms and depressive symptoms. Thus, it could be argued that our findings suggesting comparable levels of depressive symptoms among the SZ+, SAD and BPD+, which all differed from the SZ− group, is related to a tendency to under-diagnose negative symptoms and over-diagnose depressive symptoms in patients with a history of affective disturbance. Given the inverse relationship between negative and depressive symptoms in the present study (r = −0.23; p<0.0001), this interpretation cannot be ruled out. However, if the classification of depressive symptoms was merely an artifact of the classification of negative symptoms and vice versa, it is likely that we would have found that the lifetime history of negative symptoms was comparable across the SZ+, SAD and BPD+ diagnostic groups. To the contrary, we found that lifetime severity of negative symptoms significantly differed between all of these groups.

Comparison of groups on our putative measure of cognitive decline indicated that the cognitive deterioration across the course of illness was significantly worse for the SAD group than the BP+ group, with the SZ− and SZ+ group showing a decline in cognitive functioning intermediate to BPD+ and SAD. Because these analyses covaried for differences in demographic characteristics and lifetime symptomatology, this finding cannot be attributed to differences in these factors across diagnostic groups. Although it might be argued that the diagnostic groups show different levels of decline as a result of differences in level of functioning at the time of testing, the GAF score, which represents level of functioning at time of assessment, was also included as a covariate in these analyses. Moreover, these results do not appear to be driven by a single cognitive domain as the patterns of raw scores across diagnostic groups are consistent across most domains. These data are shown in Table 3. To our knowledge the present analysis represents the first data to assess cognitive decline across the spectrum of diagnoses from SZ to psychotic BPD.

**Table 3:** Mean raw scores and standard errors by diagnostic group on all cognitive tests used to calculate general cognitive ability [g].

	SZ−	SZ+	SAD	BPD+
**Digit Span**	12.24 (0.28)	12.09 (0.31)	12.22 (0.41)	13.59 (0.32)
**CVLT**	33.15 (0.75)	35.42 (0.83)	38.56 (1.14)	41.96 (0.94)
**COWAT**	30.38 (0.68)	34.06 (1.15)	31.49 (1.40)	32.46 (0.90)
**Fluency**	15.02 (0.34)	16.78 (0.38)	16.22 (0.55)	18.32 (0.48)
**Trails A (Time)**	49.14 (1.57)	45.90 (1.93)	45.17 (1.87)	42.29 (2.51)
**Trails B (Time)**	147.18 (4.93)	141.71 (5.71)	141.98 (7.67)	109.14 (5.28)

See text for additional details. Groups include: schizophrenia without mood syndrome [SZ−]; schizophrenia with superimposed mood syndrome [SZ+]; schizoaffective disorder [SAD]; bipolar disorder with psychotic features [BPD+].

It should be noted that our SAD group was considerably smaller than the other groups we assessed. Although our analyses indicated that we were sufficiently powered to detect differences amongst the groups with all preliminary and primary analyses carried out with >85% power, it is possible that the differences in sample size may have had an impact on the overall stability of the results. Finally, our data are limited by collection of clinical and neurocognitive data at a single time point, although determination of lifetime clinical ratings was bolstered by availability of substantial chart histories. Nevertheless, the convergence of these results suggests that the identification of differences in longitudinal clinical and neurocognitive profiles in patients suffering from a range of psychotic illnesses is possible. The identification of such differences may provide insight into the pathology underlying these illnesses. Moreover, the present results suggest that SAD may represent a disease entity that is distinct from both SZ and BPD. Future prospective field trials will be needed to provide longitudinal validation of the schizoaffective diagnosis as a useful clinical entity.
